# ER Quality Control Components UGGT and STT3a Are Required for Activation of Defense Responses in *Bir1-1*

**DOI:** 10.1371/journal.pone.0120245

**Published:** 2015-03-16

**Authors:** Qian Zhang, Tongjun Sun, Yuelin Zhang

**Affiliations:** Department of Botany, University of British Columbia, Vancouver, Canada; Texas A&M University, UNITED STATES

## Abstract

The receptor-like kinase SUPPRESSOR OF BIR1, 1 (SOBIR1) functions as a critical regulator in plant immunity. It is required for activation of cell death and defense responses in Arabidopsis *bak1-interacting receptor-like kinase 1*,*1* (*bir1-1*) mutant plants. Here we report that the ER quality control component UDP-glucose:glycoprotein glucosyltransferase (UGGT) is required for the biogenesis of SOBIR1 and mutations in *UGGT* suppress the spontaneous cell death and constitutive defense responses in *bir1-1*. Loss of function of *STT3a*, which encodes a subunit of the oligosaccharyltransferase complex, also suppresses the autoimmune phenotype in *bir1-1*. However, it has no effect on the accumulation of SOBIR1, suggesting that additional signaling components other than SOBIR1 may be regulated by ER quality control. Our study provides clear evidence that ER quality control play critical roles in regulating defense activation in *bir1-1*.

## Introduction

Eukaryotic cells have evolved several quality control mechanisms to monitor the folding of secretory proteins in the endoplasmic reticulum (ER) [1,2]. Correctly folded proteins are allowed to export to their final destinations, whereas misfolded proteins are retained in the ER for additional folding process or degraded by the ER-associated degradation pathway.

One of the well-studied protein folding pathway specific for secreted glycoproteins is the calnexin (CNX)/calreticulin(CRT) cycle, which involves ER-localized lectin-like chaperones CNX/CRT and the UDP-glucose:glycoprotein glucosyltransferase (UGGT) [3]. Following protein translation, preassembled glycan chains (Glc_3_Man_9_GlcNAc_2_) are transferred to the Asn (N)-residues in the N-X-Ser/Thr sequences in acceptor proteins by the oligosaccharyltransferase (OST) complex. Trimming of two glucose residues from the glycan chain by glucosidases generates proteins with monoglucosylated glycans (GlcMan_9_GlcNAc_2_), which CNX and CRT interact with and assist with folding in the ER. Subsequent removal of the remaining glucose from GlcMan_9_GlcNAc_2_ leads to dissociation of the client protein from CNX and CRT. Proteins that attained their nature structure can then enter the secretory process, whereas improperly folded proteins are recognized by UGGT and a glucose residue is added back to the Man_9_GlcNAc_2_ by the enzyme. The monoglucosylated proteins subsequently associate with CNX and CRT to go through another round of folding.

UGGT and CRT3 have been shown to play important roles in the biogenesis of transmembrane receptors in plants. Retention of the defective brassinosteroid receptor bri1–9 protein in the ER requires both UGGT and CRT3 [4,5]. In Arabidopsis *uggt* and *crt3* mutant plants, accumulation of the receptor-like kinase (RLK) EFR, which recognizes bacterial EF-Tu, is reduced [6,7]. Expression of the tobacco protein INDUCED RECEPTOR-LIKE KINASE was also shown to be dependent on NbCRT3 [8]. In tomato, silencing of CRT3a affects the biogenesis of Cf-4 and leads to loss of pathogen resistance mediated by Cf-4 [9]. In addition, loss of function mutations in Arabidopsis *STT3a*, which encodes the catalytic subunit of the OST complex, also cause reduced EFR protein level and impair its function in plant immunity [7,10].

In Arabidopsis, BAK1-INTERACTING RECEPTOR-LIKE KINASE 1 (BIR1) negatively regulates cell death and defense responses mediated by the RLK SOBIR1 (SUPPRESSOR OF BIR1, 1) [11]. Previous studies showed that activation of defense responses in *bir1–1* is also dependent on the β and γ subunits of heterotrimeric G protein as well as several ER quality control (ER-QC) components including CRT3, ERdj3b and SDF2 [12,13]. Here we report that additional ER-QC regulators, UGGT and STT3a, also play important roles in the regulation of defense responses in *bir1–1*.

## Results and Discussion

### Identification and characterization of *sobir6–1 bir1–1 pad4–1*

**Fig 1:**
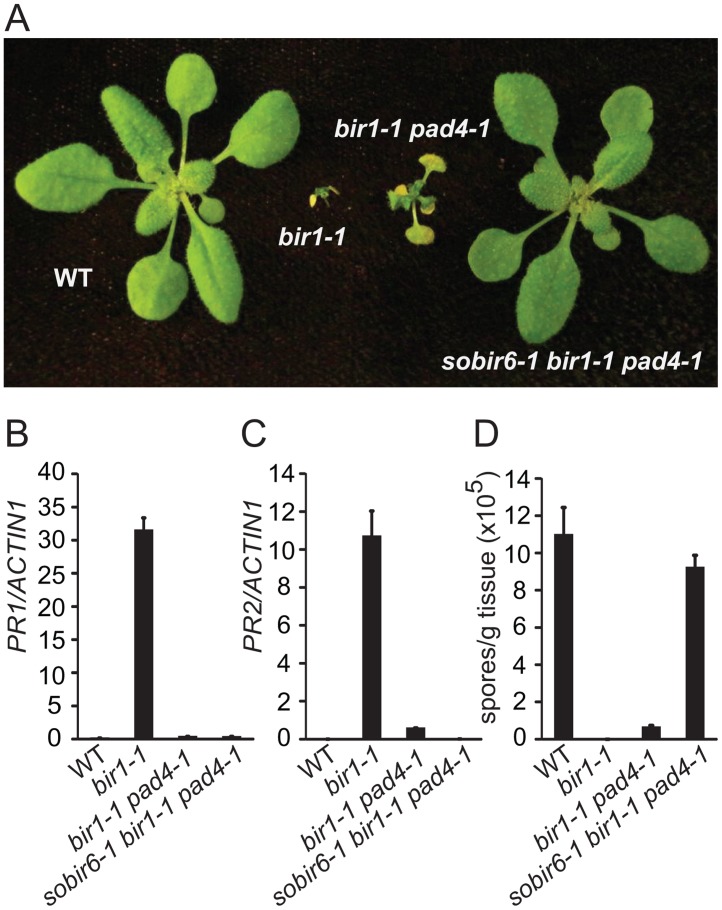
Characterization of the *sobir6–1 bir1–1 pad4–1* triple mutant. (A) Morphology of Col-0 wild type (WT), *bir1–1*, *bir1–1 pad4–1* and *sobir6–1 bir1–1 pad4–1*. Plants were grown on soil at 23°C and photographed three weeks after planting. (B-C) Expression levels of *PR1* (B) and *PR2* (C) in WT, *bir1–1*, *bir1–1 pad4–1* and *sobir6–1 bir1–1 pad4–1* seedlings compared to *ACTIN1*. Total RNA was extracted from 12-day-old seedlings grown on half-strength MS plates. (D) Growth of *H*. *a*. Noco2 on WT, *bir1–1*, *bir1–1 pad4–1* and *sobir6–1 bir1–1 pad4–1* seedlings. Error bars in (B-D) represent standard deviations of three measurements.

To identify defense pathways activated in *bir1–1*, a suppressor screen was carried out in the *bir1–1 pad4–1* mutant background [11]. *sobir6–1* is one of the mutants identified from the screen. In the *sobir6–1 bir1–1 pad4–1* triple mutant, the dwarf morphology of *bir1–1 pad4–1* is almost completely suppressed (Fig. 1A). Analysis of the expression levels of defense marker genes *PATHOGENESIS-RELATED 1* (*PR1*) (Fig. 1B) and *PR2* (Fig. 1C) in *sobir6–1 bir1–1 pad4–1* showed that *PR2* expression is significantly lower than in *bir1–1 pad4–1*. In addition, *sobir6–1 bir1–1 pad4–1* supports much higher growth of the oomycete pathogen *Hyaloperonospora arabidopsidis* (*H*.*a*.) Noco2 than *bir1–1 pad4–1* (Fig. 1D). These data indicate that the constitutive defense responses observed in *bir1–1 pad4–1* is largely suppressed by *sobir6–1*.

### *SOBIR6* encodes UGGT

**Fig 2:**
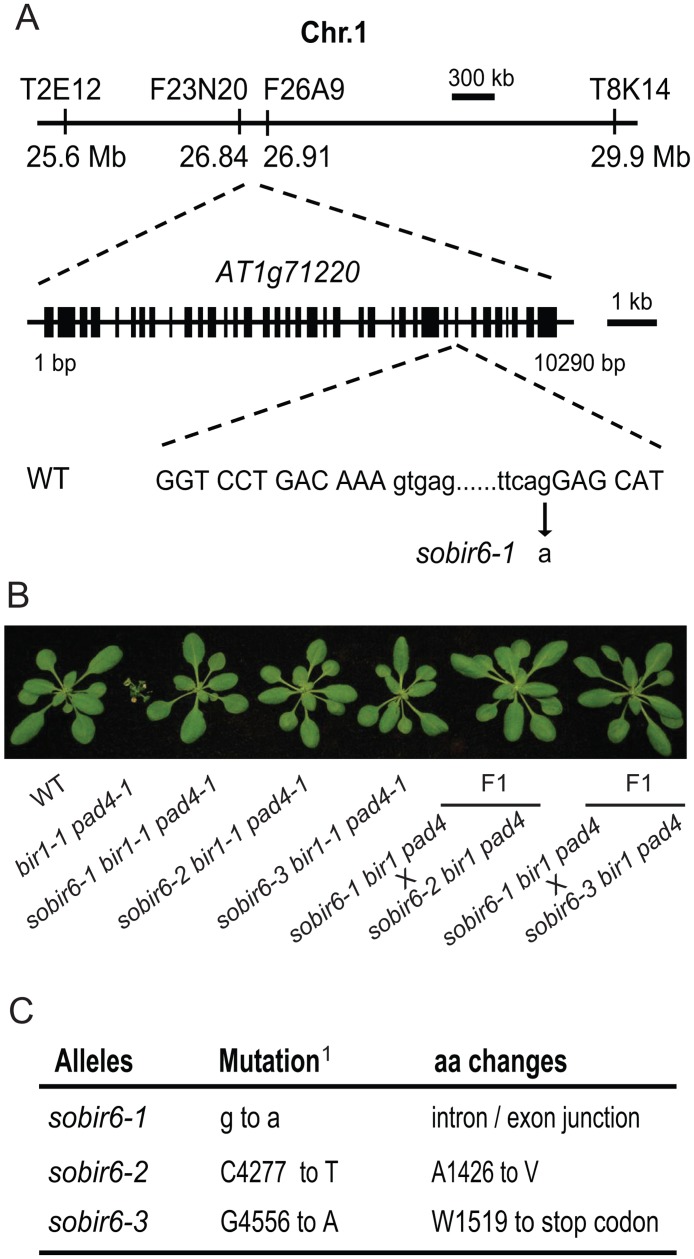
Map- based cloning of *SOBIR6*. (A) Mapping of the *sobir6–1* mutation. Positions of the mapping markers, the gene structure of *SOBIR6* and the mutation site in *sobir6–1* are shown. The exons are indicated with boxes and introns with lines. The mutation site is located at the junction between the 29th intron and 30th exon. The lower case letters represent nucleotides in the intron and the uppercase letters represent nucleotides in the exon. (B) Morphology of *sobir6 bir1–1 pad4–1* alleles and representative F1 plants of indicated crosses for the complementation test. Plants were grown on soil at 23°C and photographed three weeks after planting. (C) Mutations identified in the *sobir6* alleles. aa, amino acid. ^1^The positions of mutated nucleotide in the coding sequence are listed.

The *sobir6–1* mutation was mapped to a region between marker T2E12 and T8K14 on Chromosome 1 using a mapping population generated by crossing *sobir6–1 bir1–1 pad4–1* (in the Columbia ecotype background) with Landsberg. Further fine mapping analysis narrowed the mutation to a 70 kb region between markers F23N20 and F26A9 (Fig. 2A). To identify the *sobir6–1* mutation, PCR fragments covering this region were amplified from genomic DNA of *sobir6–1 bir1–1 pad4–1* and sequenced. A single G to A mutation was identified in *AT1G71220*, which encodes the ER-QC component UGGT. The mutation is located at the junction between the 29^th^ intron and 30^th^ exon of *UGGT*.

In the same mutant screen, we also identified two additional alleles of *sobir6*. Both of them failed to complement *sobir6–1* (Fig. 2B). Sequence analysis of *UGGT* in *sobir6–2* and *sobir6–3* showed that they also contain mutations in the gene. In *sobir6–2*, a C to T mutation changes Ala_1426_ to Val. In *sobir6–3*, a G to A mutation introduces a stop codon in the coding region (Fig. 2C). These data suggest that *SOBIR6* is *UGGT*.

### UGGT is required for constitutive defense responses in *bir1–1*

**Fig 3:**
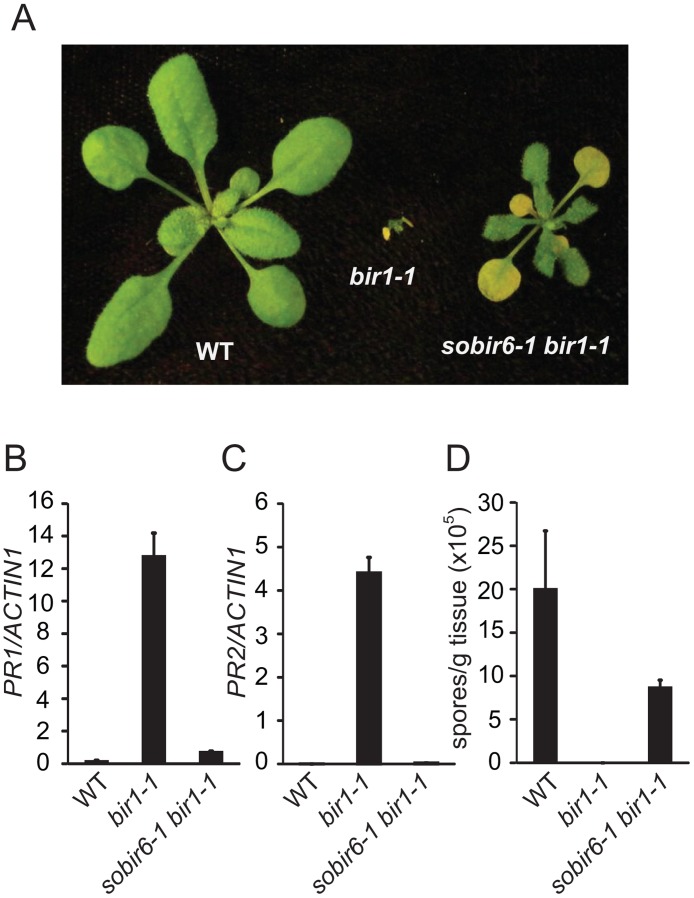
Characterization of the *sobir6–1 bir1–1* double mutant. (A) Morphology of wild type (WT), *bir1–1* and *sobir6–1bir1–1* plants. Plants were grown on soil at 23°C and photographed three weeks after planting. (B-C) Expression levels of *PR1* (B) and *PR2* (C) in WT, *bir1–1* and *sobir6–1 bir1–1* seedlings as normalized with *ACTIN1*. Total RNA was extracted from 12-day-old seedlings grown on half-strength MS plates. (D) Growth of *H*. *a*. Noco2 on WT, *bir1–1* and *sobir6–1 bir1–1* seedlings. Error bars in (B-D) represent standard deviations of three measurements.

To test whether *sobir6–1* can suppress the constitutive defense responses in *bir1–1* in the absence of *pad4–1*, we isolated the *sobir6–1 bir1–1* double mutant from the F2 population of a cross between *sobir6–1 bir1–1 pad4–1* and wild type. *sobir6–1 bir1–1* is much bigger than *bir1–1*, but smaller than wild type (Fig. 3A). In *sobir6–1 bir1–1*, expression of both *PR1* and *PR2* is greatly reduced compared to that in *bir1–1* (Fig. 3B-C). As shown in Fig. 3D, resistance to *H*.*a*. Noco2 is also considerably reduced in *sobir6–1 bir1–1*. These data suggest that UGGT is required for the constitutive defense responses in *bir1–1*. This is consistent with the requirement of another component of the CNX/CRT cycle, CRT3, for the autoimmune phenotype in *bir1–1* [13].

### The autoimmune phenotype of *bir1–1* is partially suppressed by *stt3a-2*

**Fig 4:**
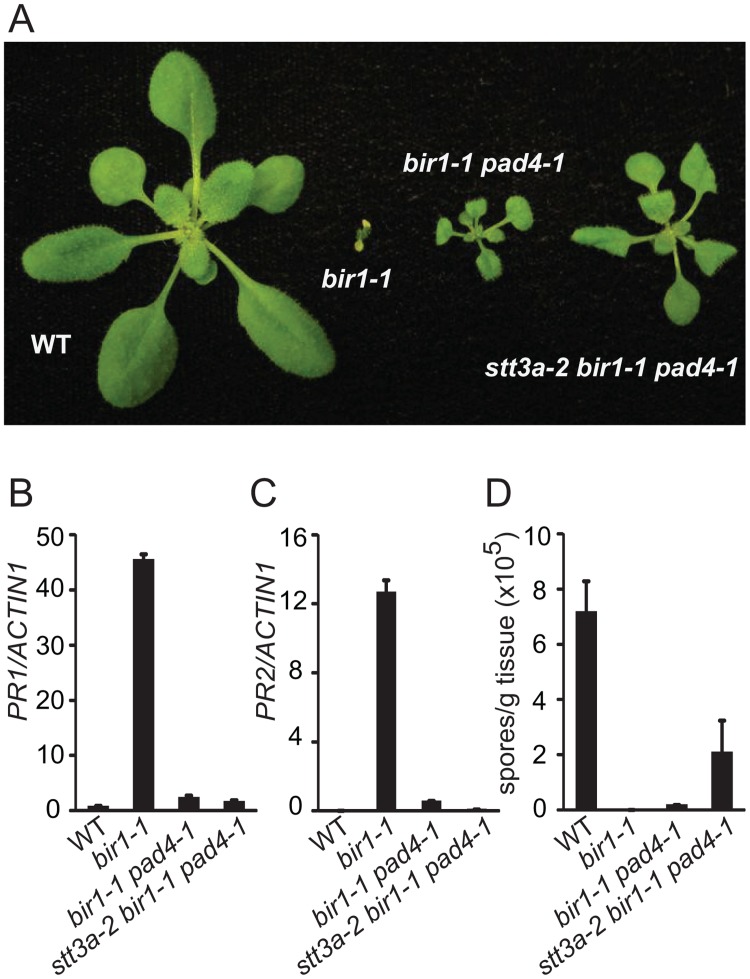
Characterization of the *stt3a-2 bir1–1 pad4–1* triple mutant. (A) Morphology of wild type (WT), *bir1–1*, *bir1–1 pad4–1* and *stt3a-2 bir1–1 pad4–1* plants. Plants were grown on soil at 23°C and photographed 3 weeks after planting. (B-C) Expression levels of *PR1* (B) and *PR2* (C) in WT, *bir1–1*, *bir1–1 pad4–1* and *stt3a-2 bir1–1 pad4–1* seedlings as normalized with *ACTIN1*. Total RNA was extracted from 12-day-old seedlings grown on half-strength MS plates. (D) Growth of *H*. *a*. Noco2 on WT, *bir1–1*, *bir1–1 pad4–1* and *stt3a-2 bir1–1 pad4–1* seedlings. Error bars in (B-D) represent standard deviations of three measurements.

**Fig 5:**
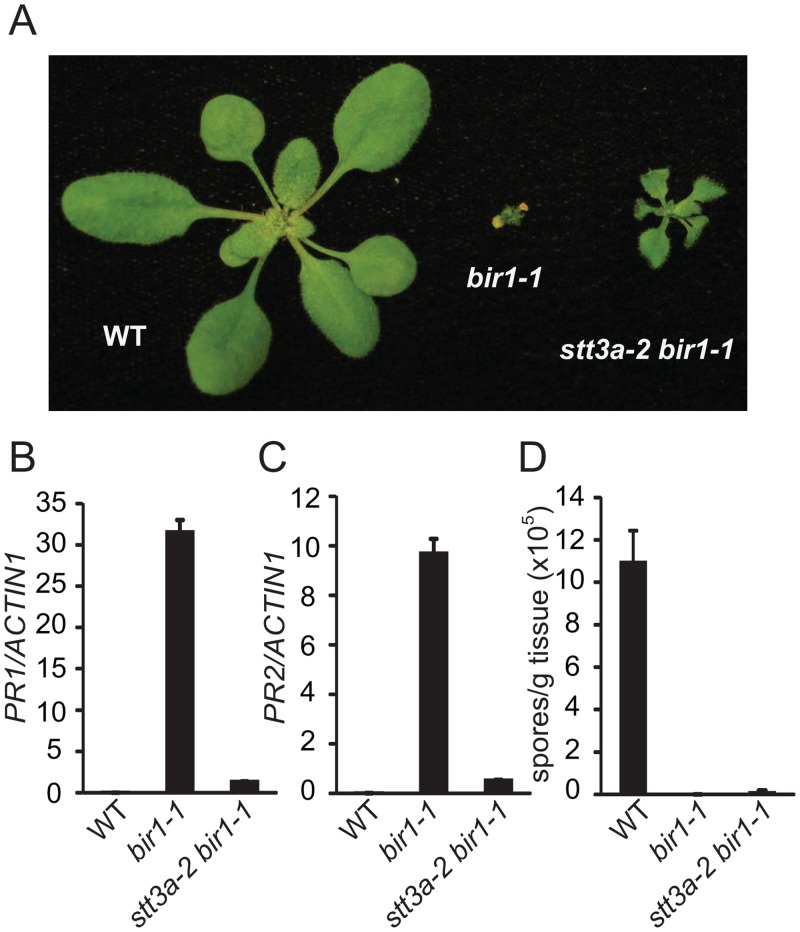
Characterization of the *stt3a-2 bir1–1* double mutant. (A) Morphology of wild type (WT), *bir1–1* and *stt3a-2 bir1–1*. Plants were grown on soil at 23°C and photographed three weeks after planting. (B-C) Expression levels of *PR1* (B) and *PR2* (C) in WT, *bir1–1* and *stt3a-2 bir1–1* seedlings normalized by *ACTIN1*. Total RNA was extracted from 12-day-old seedlings grown on half-strength MS plates. (D) Growth of *H*. *a*. Noco2 on WT, *bir1–1* and *stt3a-2 bir1–1* seedlings. Error bars in (B-D) represent standard deviations of three measurements.

Since STT3a is involved in co-translational N-glycosylation of nascent proteins before they enter the CNX/CRT cycle [3], we tested whether STT3a is required for the constitutive defense responses in *bir1–1* by crossing *stt3a-2* with *bir1–1 pad4–1* and isolating the *stt3a-2 bir1–1 pad4–1* triple mutant and the *stt3a-2 bir1–1* double mutant in the F2 generation. As shown in Fig. 4A, *stt3a-2 bir1–1 pad4–1* is larger than *bir1–1 pad4–1*, but considerably smaller than wild type. In *stt3a-2 bir1–1 pad4–1*, the expression of both *PR1* and *PR2* is lower than in *bir1–1 pad4–1* (Fig. 4B-C). *H*.*a*. Noco2 growth on *stt3a-2 bir1–1 pad4–1* is much higher than on *bir1–1 pad4–1*, but significantly lower than on wild type (Fig. 4D). The *stt3a-2 bir1–1* double mutant retained the dwarf morphology of *bir1–1*, but is larger in size (Fig. 5A). In *stt3a-2 bir1–1*, the expression of both *PR1* and *PR2* is greatly reduced compared to that in *bir1–1* (Fig. 5B-C). There is a small amount of *H*.*a*. Noco2 growth on the *stt3a bir1–1* double mutant compared to almost no growth of the pathogen on *bir1–1* plants (Fig. 5D). Taken together, the autoimmune phenotype of *bir1–1* is partially dependent on STT3a. Our data suggest that STT3a-dependent N-glycosylation is also critical for activation of defense responses in *bir1–1*.

### *sobir6–1* affects the protein level of SOBIR1

**Fig 6:**
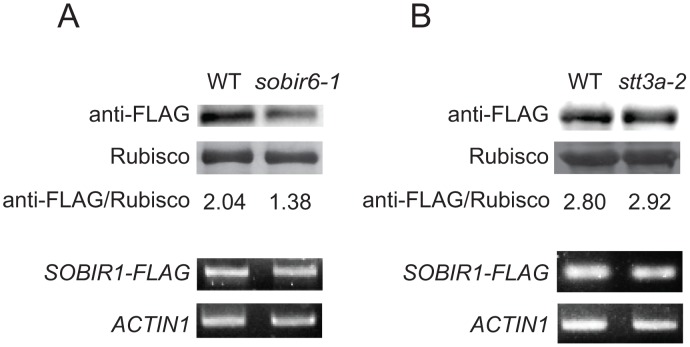
Accumulation of SOBIR1 protein in *sobir6–1* and *stt3a-2*. (A) Western blot analysis of SOBIR1-FLAG protein level (top) and RT-PCR analysis of *SOBIR1-FLAG* expression (bottom) in wild type (WT) and *sobir6–1* mutant background. (B)Western blot analysis of SOBIR1-FLAG protein level (top) and RT-PCR analysis of *SOBIR1-FLAG* expression (bottom) in WT and *stt3a-2* mutant background. Protein and RNA samples were extracted from 12-day-old seedlings grown on half-strength MS plates at 23°C. Rubisco was used as protein loading control and *ACTIN1* was used as RNA control.

Because the constitutive defense responses in *bir1–1* are dependent on the RLK SOBIR1 and the accumulation of SOBIR1 is dependent on the ER-QC component CRT3 [13], we further tested whether UGGT and STT3a are also required for SOBIR1 accumulation. A transgenic line expressing the SOBIR1-FLAG fusion protein under its own promoter in wild type background was crossed into *sobir6–1* or *stt3a-2*, a T-DNA knockout mutant of *STT3a* As shown in Fig. 6A, the SOBIR1-FLAG protein level is considerably lower in *sobir6–1* than in wild type background, suggesting that UGGT is also required for the accumulation of SOBIR1-FLAG protein. In contrast, the SOBIR1-FLAG protein levels are similar in *stt3a-2* and wild type background.

In addition to its role in cell death and defense activation in *bir1–1*, increasing evidences suggest that SOBIR1 functions as a critical component of receptor-like protein (RLP)-mediated immunity [14,15]. SOBIR1 proteins in tomato interact with two RLPs Cf-4 and Ve1 and are required for Cf-4 and Ve1 mediated immunity. In addition, SOBIR1 functions together with Arabidopsis RLP30 in defense against necrotrophic fungi [16].

Our study provided additional evidence that ER-QC plays important roles in the biogenesis of SOBIR1 and reduced accumulation of SOBIR1 contributes to the suppression of *bir1–1* mutant phenotypes by mutations in *UGGT*. Because biogenesis of SOBIR1 in Arabidopsis is dependent on multiple components of ER-QC, it is likely that accumulation of SOBIR1 proteins in tomato also relies on ER-QC. The compromised Cf-4 and Ve1-mediated immune responses observed in tomato plants when CRT3a was silenced [9,17] might be partially due to reduced accumulation of tomato SOBIR1.

Compared to almost complete suppression of the autoimmune phenotype in *bir1–1 pad4–1* by *uggt* and *crt3* mutants, *stt3a-2* has a much smaller effect on the morphology as well as defense responses in *bir1–1 pad4–1*. This can probably be explained by genetic redundancy. In Arabidopsis, there is a close homolog of STT3a named STT3b [18]. It is likely that STT3b can partially compensate the loss of the function of STT3a in N-glycosylation.

As SOBIR1 accumulation is not affected in *stt3a-2*, the mechanism of how *stt3a-2* suppresses the phenotypes of *bir1–1* remains to be determined. It is possible that the contribution of STT3a to the biogenesis of SOBIR1 is masked by genetic redundancy between STT3a and STT3b. Since SOBIR1 usually functions together with RLPs, it is likely that one or more RLPs might be involved in the activation of cell death and defense responses in *bir1–1* and the suppression of *bir1–1* mutant phenotypes by *stt3a-2* might be caused by reduced accumulation of the RLPs. Previously UGGT and STT3a were also shown to be required for SA-induced defense responses [7]. It is possible that reduced response to SA also contributes to the suppression of *bir1–1* mutant phenotypes by *stt3a-2*.

## Method

### Plant material

*bir1–1*, *bir1–1 pad4–1* and the identification of suppressor mutants of *bir1–1 pad4–1* have been described previously [11]. *stt3a-2* (SALK_058814) was obtained from the Arabidopsis Biological Resource Center and has been described previously [18]. All plants were grown at 23°C under 16h light/8h dark. To isolate the *sobir6–1* single mutant and *sobir6–1 bir1–1* double mutant, *sobir6–1 bir1–1 pad4–1* was crossed with a Col-0 plant. In F2, *sobir6–1* single mutant and *sobir6–1 bir1–1* double mutant were isolated by PCR genotyping. To generate *stt3a-2 bir1–1* double mutant and *stt3a-2 bir1–1 pad4–1* triple mutant, *stt3a-2* was crossed with *bir1–1 pad4–1*. *stt3a-2 bir1–1* and *stt3a-2 bir1–1 pad4–1* were identified in F2 by PCR genotyping.

### Mutant characterization

*H*. *a*. Noco2 infection was performed on 12-day-old seedlings. The seedlings were sprayed with spore suspension at a concentration of 50,000 spores per ml water. Sprayed plants were covered with a clear dome and kept at 16°C under 12h light/12h dark cycles in a growth chamber. The humility in the growth chamber was approximately 95%. Infection results were scored seven days later as previously described [19].

For gene expression analysis, RNA was extracted from 12-day-old seedlings grown on half-strength MS plates using EZ-10 Spin Column Plant RNA Mini-Preps Kit from Bio Basic Inc. About six seedlings were collected and extracted in each sample. The extracted RNA was reverse transcribed into total cDNA using Easy Script Reverse Transcriptase from *Applied Biological Materials* Inc. Real- time PCR was performed in triplicate with three independent RNA samples using SYBR Premix Ex Taq IIfrom Takara. Total cDNA was used as a template to determine the expression level of target genes with *ACTIN1* as control. Primers used for real-time PCR analysis of *ACTN1*, *PR1* and *PR2* have been described previously [20].

### Analysis of SOBIR1 protein level in *sobir6–1* and *stt3a-2*

A transgenic line expressing the SOBIR1 protein with a 3xFLAG tag [13] was crossed with *sobir6–1* and *stt3a-2*. In F2, plants that were homozygous for *sobir6–1* and *stt3a-2* and carried the *SOBIR1-FLAG* transgene were identified by PCR. For Western blot analysis, about 20 seedlings from half-strength MS plate were collected in liquid nitrogen for each sample. The samples were ground and boiled in 2×SDS gel-loading buffer (100mM Tris-Cl pH6.8, 4% w/v sodium dodecyl sulfate, 0.2% bromophenol blue, 20% w/v glycerol, 200mM DTT). Supernatants were subject to Western blot analysis using the anti-flag M2 antibody (Sigma-Aldrich).

## Supporting Information

S1 Table Sequences of markers used in this project. (PDF)
