# Supplementary material for: ER Quality Control Components UGGT and STT3a Are Required for Activation of Defense Responses in Bir1-1
Source: PLoS One. 2015 Mar 16;10(3):e0120245. doi: 10.1371/journal.pone.0120245 (PMC4361565; doi:10.1371/journal.pone.0120245)
Supplement: S1 Table — (PDF) [file pone.0120245.s001.pdf]

Table S1. Sequences of markers used in this project.

Marker name	Position (MB)	Polymorphism type	Primer 1	Primer 2
T2E12	Chr.1-25.66	Indel	F: tggtgttataatcatgaagc	R: gtgttccattttggtacttag
F23N20	Chr.1-26.84	Indel	F: tctgagtagttgtgtcgcac	R: attgggtagtatccattatg
F26A9	Chr.1-26.91	Indel	F: gaatatcgttggctataagg	R: tgcacccatcagaggagttc
T8K14	Chr.1-29.87	Indel	F: gacaagaacctcataccttgc	R: caagtgatggagaagctgtc
